# Morphological and molecular characterization of *Fusarium* spp. associated with olive trees dieback in Tunisia

**DOI:** 10.1007/s13205-016-0587-3

**Published:** 2017-04-11

**Authors:** Rahma Trabelsi, Hanen Sellami, Yâakoub Gharbi, Samira Krid, Manel Cheffi, Sonia Kammoun, Mariem Dammak, Aymen Mseddi, Radhouane Gdoura, Mohamed Ali Triki

**Affiliations:** 1 Laboratoire d’Amélioration et Protection des Ressources Génétique de l’Olivier, Institut de l’Olivier, Université de Sfax, BP1078, Sfax, Tunisia; 2 Laboratoire de Recherche Toxicologie-Microbiologie Environnementale et Santé (LR17ES06), Faculté des Sciences de Sfax, Université de Sfax, Sfax, Tunisia

**Keywords:** Disease symptoms, *Fusarium* spp., Molecular identification, Olive tree (*Olea europaea*), Pathogenicity

## Abstract

Dieback and wilting symptoms caused by complex soilborne fungi are nowadays the most serious threatening disease affecting olive trees (*Olea europaea*) in Tunisia and presumably in many Mediterranean basin countries*. Fusarium* is one of the important phytopathogenic genera associated with dieback symptoms of olive trees. The objective of the present study was to confirm the pathogenicity of *Fusarium* spp. isolated from several olive-growing areas in Tunisia. According to the pathogenic test done on young olive trees (cv. Chemlali), 23 out of 104 isolates of *Fusarium* spp. were found to be pathogenic and the others were weakly or not pathogenic. The pathogenic *Fusarium* spp. isolates were characterized using molecular methods based on ITS PCR. Isolation results revealed the predominance of *Fusarium solani* (56.5%) and *F. oxysporum* species (21.7%) compared to *F. chalmydosporum* (8.7%), *F. brachygibbosum* (8.7%) and *F. acuminatum* (4.34%). Based on pathogenicity test, disease severity was highly variable among the 23 pathogenic isolates tested (*P* < 0.05) where *F. solani* was the most aggressive dieback agent. To the best of our knowledge, this is the first work that shows that *Fusarium* spp. might be a major agent causing dieback disease of olive trees in Tunisia.

## Introduction

Olive tree (*Olea europaea*) is the most widely cultivated tree species in Tunisia. With a total cultivated area of about 1.7 million ha and more than 70 million olive trees (anonymous), Tunisia is ranked as the fourth producer of olive oil worldwide. However, many insect pests and plant pathogens constantly threaten olive cultivation. The dieback and wilting symptoms induced by complex soilborne fungi has caused considerable economic losses in olive orchards in Tunisia (Boulila and Mahjoub 1994; Triki et al. 2006, 2009, 2011; Gharbi et al. 2014, 2015). Among these fungi, *Fusarium* species may cause disease in olive trees. Diagnosis of dieback and decline of young olive trees revealed the presence of a broad range of fungi isolated from the affected plant tissues (Triki et al. 2009). Some species are known as common pathogens on olive trees, such as *Verticillium dahliae* (*V. dahliae*), but also an increased frequency of isolates belonging to the genus *Fusarium* was noted. These observations together with the increase of inquiries received by the Olive Tree Institute of Tunisia, led us to investigate the etiology of this disease, to perform pathogenicity tests of the different *Fusarium* species recovered from olive orchards and their identification based on morphological and molecular features. Disease incidence has increased over years and seems to be linked to changes in farming systems and the subsequencalculated using the following formulat decline in soil fertility. Regarding olive production, some fluctuations have been recorded which were probably linked to climatic conditions and to the presence of some fungi especially in soils previously cultivated with some susceptible vegetable crops (potato, tomato, pepper, eggplant, etc.). Consequently, with the establishment of new plantations, incidence of wilting, dieback and death of young trees has also increased (Triki et al. 2006, 2009, 2011). Disease impact was serious in nurseries and orchards during the first years after establishment, especially during spring and summer, causing important damage to new plantations. However, root rot of young olive trees was reported to be an emerging problem in nurseries and some important olive grove areas worldwide (Al-Ahmed and Hamidi 1984; Boulila et al. 1992; Sanchez-Hernandez et al. 1998; Lucero et al. 2006). *Fusarium* species have been recorded from several parts of the world and they are known to be pathogenic to many plants (Boughalleb et al. 2005; Mehl and Epstein 2007). In Tunisia, Ayed (2005) demonstrated the pathogenicity of *Fusarium* spp. on potato plants. Despite the importance of the olive-growing sector in Tunisia, this area is threatened by different species of soilborne pathogenic fungi, such as *Fusarium*. All these observations have raised the attention of researcher about the possible involvement of new pathogenic forms that may have evolved from other species infecting Solanaceous crops. Such question could be answered by investigating the prevalence and pathogenicity of each isolated fungal species. This will provide useful information towards a better understanding of the current phytosanitary status of olive trees, thus contributing to the design of better disease management programs in these infected areas. Therefore, the present study aims to (1) characterize morphologically and molecularly by polymerase chain reaction-internal transcribed spacer (ITS) *Fusarium* spp. isolates recovered from infected olives plants; (2) assess their pathogenicity and report the role of some abiotic factors in the increase of root rot disease.

## Materials and methods

### *Fusarium* isolates and culture conditions

One hundred and four isolates of *Fusarium* were obtained in 2011 from 65 roots and 114 stems belonging to diseased samples of olive trees showing typical wilt symptoms removed from many important olive-growing regions in Tunisia, including governorates of Monastir (Sahline, Boumerdes, Zarmedine), Kasserine, Sousse (Chott-Mariem), Sfax (Sidi-Bouakkazine), Mahdia, Sidi-Bouzid (Souk-jdid), Zaghouan (Nadhour) and Kairouan (Chbika) (Table 1). Samples were collected from more than 100 orchards and carried separately to the laboratory for isolation. Infected plant tissues were rinsed twice in water and then disinfected with 5% sodium hypochlorite for 5 min, washed with sterilized distilled water, prior to be cut aseptically into 0.5–2 cm pieces. Four to five pieces from each sample were placed onto each potato dextrose agar (PDA; Difco) plate. After 1 week of incubation at 25 °C, isolates were identified on the basis of their cultural characteristics and the morphology of their vegetative and reproductive structures produced on different culture media according to different keys of identifications (Booth 1966; Dhingra and Sinclair 1978; Nelson et al. 1983; Agrios 2004; Nasraoui 2006). Fungal isolates derived from single-spore cultures were obtained using the serial spore dilution method (Choi et al. 1999). Briefly, 1 ml of each serial dilution was mixed with melted nutrient agar and transferred to sterile plates. The plates were incubated at 25 °C in the dark and then visualized under the microscope to observe the germinating spores. These were later cut from the medium and transferred to new PDA plates for development of colonies. The pure cultures were maintained on PDA slants at 4 °C. The isolates were cultured in potato dextrose broth (PDB; Difco) on a rotary shaker (150 rpm) at 25 °C. The fungal mycelium was recovered by filtration, lyophilized, and stored at −20 °C until use.

**Table 1:** List of *Fusarium* species isolated from olive plants collected from different sampling sites in Tunisia; (−) absent in the Solanaceous crops

Isolates code	Molecular identification	Sampling site	Diseased organ	Solanaceous crops	Accession number
Fso1	*F. solani*	Sfax	Collar and Stem	Potato	KU528848
Fso2	*F. solani*	Sfax (Sidi Bou Akkazine)	Root	Pepper	KU528850
Fso3	*F. solani*	Monastir	Collar	Tomato	KU528851
Fso4	*F. solani*	Sfax (Mahres)	Root and Collar	Potato	KU528852
Fso5	*F. solani*	Kasserine	Collar	Potato	KU528854
Fso6	*F. solani*	Kasserine	Collar	Potato	KU528855
Fso7	*F. solani*	Monastir	Collar	Tomato	KU528857
Fso8	*F. solani*	Sousse	Collar and Stem	–	KU528858
Fso9	*F. solani*	Sfax (Mahres)	Collar	Piment	KU528859
Fso10	*F. solani*	Sfax (Mahres)	Collar	Potato	KU528860
Fso11	*F. solani*	Sousse (Sidi Bou Ali)	Collar	Potato	KU528861
Fso12	*F. solani*	Sfax (Sidi Bou Akkazine)	Collar	Potato	KU528862
Fso13	*F. solani*	Sousse	Root	Potato	KU528863
Fox1	*F. oxysporum*	Kairouan	Collar	–	KU528844
Fox2	*F. oxysporum*	Monastir	Collar	Pepper	KU528846
Fox3	*F. oxysporum*	Monastir	Collar	–	KU528847
Fox4	*F. oxysporum*	Sfax	Collar	–	KU528853
Fox5	*F. oxysporum*	Kasserine	Root and Collar	–	KU528856
Fch1	*F. chlamydosporum*	Sfax	Root and Collar	Potato	KU528845
Fch2	*F. chlamydosporum*	Sfax	Collar	–	KU528865
Fbr1	*F. brachygibbosum*	Sfax	Root	–	KU528849
Fbr2	*F. brachygibbosum*	Kairouan	Collar	–	KU528864
Fac	*F. acuminatum*	Monastir	Collar and stem	Tomato	KU528866

### Pathogenicity assay

Pathogenicity of the collected *Fusarium* spp. isolates was performed on 2-year-old olive plants (cv. Chemlali) (Rodriguez-Jurado et al. 1993; Triki et al. 2011; Gharbi et al. 2014). Fungal inoculum was prepared from 10-day-old cultures grown in PDA medium. Mycelium was recovered by aseptically scraping the surface of the medium and transferred into flasks containing sterilized distilled water. The conidial suspension was adjusted to 10^6^ conidia/ml. Plants roots were washed, dried and dipped for 1 h in the prepared conidial suspension. After inoculation, olive plants were transplanted into new polyethylene pots containing a sterile substrate (peat:sand, 1:1 v/v). The experiment was conducted in a growth chamber in the Olive Tree Institute of Tunisia. Temperature during the trial was 23 ± 2 °C and plants were maintained under high relative humidity about 96% and a 16-h photoperiod. The experiment was conducted according to a randomized complete block with six replicates per isolate and six control plants dipped in sterilized distilled water.

### Evaluation of pathogenicity

Disease severity was assessed visually and weekly, starting at 15 days post-inoculation. A scale from 0 to 4 was used based on the percentage of affected plant tissues (0 = healthy plant; 1 = ≤33% affected tissues; 2 = 34–66% affected tissues; 3 = 67–99%; 4 = dead plant). Estimation of the area under disease progress curve (AUDPC) was calculated as described previously (Campbell and Madden 1990). AUDPC values were used to classify isolates of the different *Fusarium* species. Analysis of variance was performed with a least significant difference with SPSS software (IBM Software) to determine the variability among isolates.

### Measure of radial growth rate

The fungal radial growth rate (RGR) was determined according to Lamrani (2009). Pure culture of each *Fusarium* species was placed at the center of PDA and SNA (Synthetisher Nahrst off Agar) plates (Nirenberg 1976) and incubated separately at 11, 19, 27, 40 or 45 ± 2 °C. The diameter (D) of fungal colony formed at each temperature tested was measured daily using a digital caliper. Radial growth rate was calculated using the following formula (Lamrani^2009^) where:radial growth rate (mm/day) = RGR max (D max/2)/number of days.

### DNA extraction

DNA was extracted only from 23 pathogenic *Fusarium* spp. isolates. The mycelium of each isolate was collected by scraping the surface of growing colonies on PDA medium (previously incubated for 1 week at 25 °C). After grinding 100 mg of fungal mycelia from each isolate in liquid nitrogen, the genomic DNA was extracted using the ZR Fungal/Bacterial DNA mini prep D6005 Kit (Zymo Research, Irvine, CA, USA) as recommended by the manufacturer. The DNA purity and concentration were determined using a NanoDrop ND1000 spectrophotometer (NanoDrop Technologies Inc). Aliquots of samples were also analyzed on a 1% agarose gel to check DNA quality.

### PCR amplification

All the isolates were characterized by amplification and sequencing of the ITS region. Amplification of ITS region was carried out using ITS1 and ITS4 primers (ITS1: 5′-TCCGTAGGTGAACCTGCGG/ITS4: 5′ TCCTCCGCTTATTGATATGC (White et al. 1990). PCR reactions were performed in 25 μl final volume containing 0.2 mM dNTPs mix, 1 μM of each primer, 2.5 mM MgCl_2_, 1 U Taq DNA polymerase (Promega, France) and 50 ng of fungal DNA. Optimal PCR efficacy was obtained with an initial denaturation at 95 °C for 3 min followed by 35 amplification cycles (denaturation, 95 °C for 35 s, annealing, 55 °C for 1 min, and extension, 72 °C for 2 min), and a final extension at 72 °C for 10 min. PCR products were visualized on 1.5% agarose gel along a 1-kb DNA ladder (Promega, France).The fragments were excised and further purified for sequencing. Briefly, DNA extracted using the QIAquick Gel extraction Kit (Qiagen, USA) was then subjected to cycle sequencing using the BigDye Terminator Cycle Sequencing Ready Reaction Kit (Applied Biosystems, France) and processed by the ABI 3100 Genetic Analyzer. The obtained sequences were compared with the sequences available in GenBank by using the BLAST server from the NCBI website (http://www.ncbi.nlm.nih.gov/BLAST).

### Sequencing alignment and phylogenetic analysis

The program Molecular Evolutionary Genetic Analysis software, ver. 7.0 (MEGA7.0; http://www.megasoftware.net) was used to edit and align the ITS sequences. In order to assess the relationships between the genetic traits of the identified *Fusarium* species and their pathogenicity data, the ITS sequences were used to generate phylogenetic tree grouping the isolates in major clusters. The aligned sequences were deposited in the GenBank database. In this study, phylogenetic tree was generated using maximum parsimony (MP) in MEGA5.0. Bootstrap values for the maximum parsimony tree (MPT) were calculated for 1000 replicates. The edited ITS sequences were compared with other available *Fusarium* species sequences available in the GenBank.

### Statistical analysis

All the optimization studies were conducted in triplicate and the data were analyzed using single factor analysis of variance (ANOVA). All the data were summarized as mean ± SD (standard deviation) of triplicates (*n* = 3). ANOVA was performed using Graph Pad Prism version 5.0b for Mac (Graph Pad Software, Inc., San Diego, CA, USA). Significant differences between isolates were assessed at each time point by multiple comparisons of means using the Tukey test at* P* < 0.05.

## Results

### Isolation of *Fusarium* spp.

In this study, we were only interested in isolation of *Fusarium* spp. but other soilborne pathogens may also act as fungal complex in the dieback syndrome. Isolations performed from roots, stems and collar of wilted olive plants collected from several olive-growing areas during a survey of olive diseases conducted in 2011, allowed the recovery a high frequency of *Fusarium* spp. where 104 isolates were recovered compared to the other species.

### Morphological identification and pathogenicity test of *Fusarium* spp.

One hundred and four fungal colonies formed on PDA medium were identified as *Fusarium* spp. based on the morphology of their colonies using the *Fusarium* synoptic keys for species identification of Leslie and Summerell (2006) and Nelson et al. (1983). These isolates were identified as *F. solani*, *F. oxysporum*, *F. chlamydosporum, F. acuminatum* and *F. brachygibbosum* and other *Fusarium* spp. Colonies of *F. solani* formed on PDA (Fisher et al. 1982) showed typical curved macroconidia widest in the middle of their length. The microconidia are oval, reniform, elongated oval to sometimes obovoid with a truncate base, and septated into 3–7. The size of conidia measures as: 44–78 × 3.3–5.6 μm. Chlamydospores formed were relatively abundant in mycelium, mostly globose, subglobose, intercalary and rough walled. The species are distinguished also by its white-to-cream mycelium and the production of green pigments on PDA medium. *F. oxysporum* colonies were characterized by an abundant white cottony mycelium and a dark-purple undersurface on PDA. Its microconidia are oval to ellipsoid or kidney shaped. Macroconidia were oval tapering and septated in 3 cells, their measurement indicated a size of 32–56 × 3.1–5.7 μm. Chlamydospores formed in chains. *F. brachygibbosum* showed microconidia are oval and the macroconidia are tapered and pointed, the size of conidia measures as follows: 25–68 × 3.6–6.0 μm. For *F. acuminatum*, microconidia are not a reliable taxonomic indicator, but this fungus was characterized by the long tapering curved macroconidia, with measured size of 35–58 × 3.0–5.0 μm, with the red pigmentation of the culture medium. Colonies of *F. chlamydosporum* were characterized by their white mycelial growth and abundant gamma-shaped microconidia and their curved-pointed macroconidia, measuring 37–58 × 3.0–5.0 μm. The other colonies were identified at the genus level only as *Fusarium*.

**Fig. 1:**
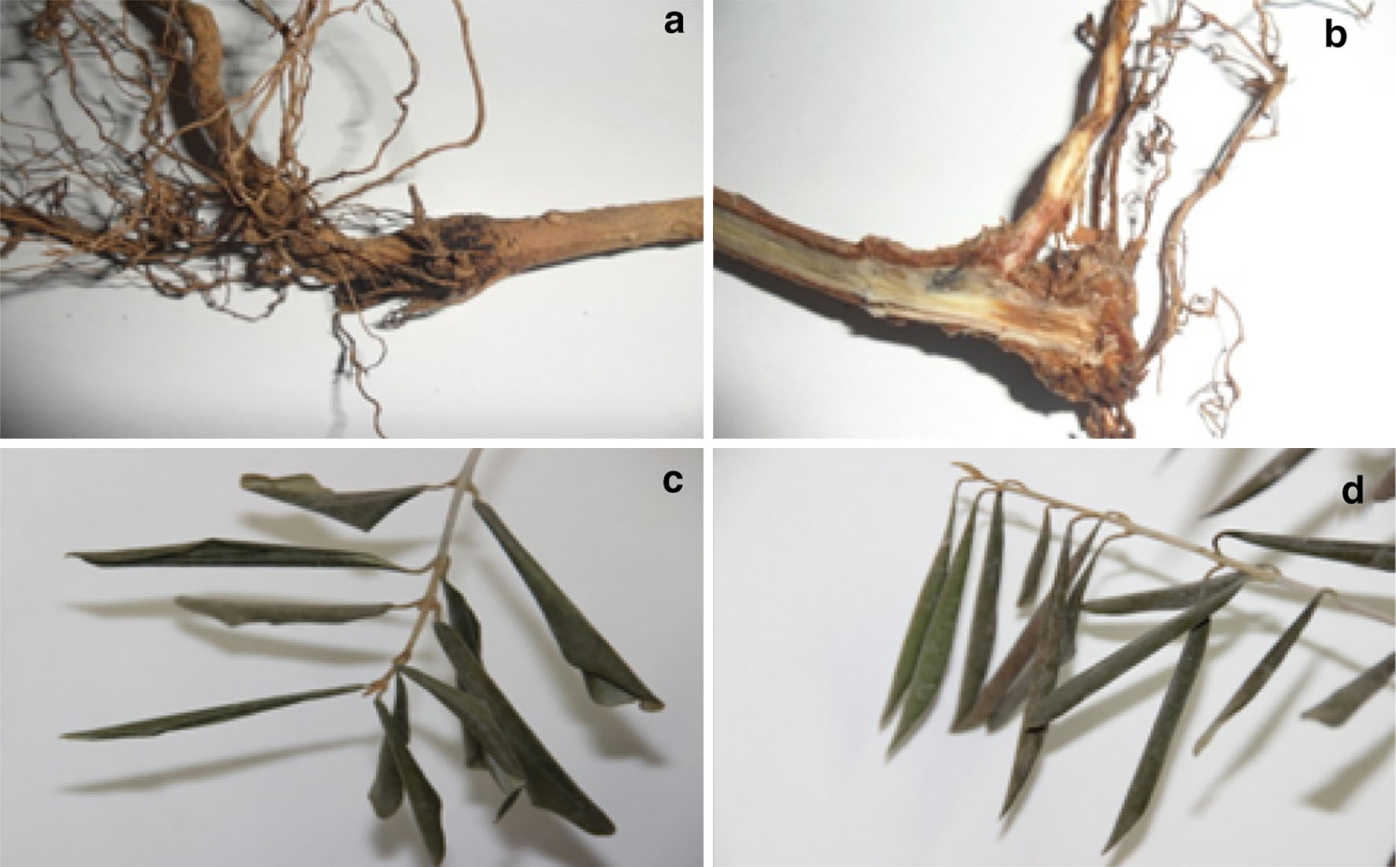
Different symptoms of inoculated olive tree by* Fusarium* isolates. **a** Brown discoloration of the root, crown and stem;** b** browning of the vascular tissues** c**–**d** winding and drying of leaves (**c**: initial stage;** d**: final stage)

In the present study, the results of the pathogenicity test revealed that only 23 isolates among a total of 104 isolates of *Fusarium* spp. were found to be pathogenic and the others were weakly or not pathogenic. The symptoms induced by pathogenic *Fusarium* isolates developed within 20–25 days after inoculation. Leaves became brown and rolled gradually inwards. Necrosis and brown discoloration were observed on roots, crown and stem as well as a browning of the vascular tissues (Fig. 1). However, no symptoms were observed on the artificially inoculated trees by some isolates of *Fusarium* spp. which were classified as not pathogenic. The inoculated fungi were consistently isolated from the diseased plants, but not from negative control plants and non-virulent isolates. Thus, the Koch’s postulates were fulfilled.

### Characterization of pathogenic *Fusarium* species

Besides the morphological identification, the identity of the 23 pathogenic *Fusarium* isolates out of the 104 collected was confirmed by sequencing the amplified fragment of internal transcribed spacer region using the ITS universal primers ITS1 and ITS4 (Table 1). The analysis of ITS sequences of these 23 isolates by BLAST have shown that 13 of them (56.5%) were affiliated to the species *F. solani* with 99% homology. The accession numbers of each ITS sequence of these 13 isolates assigned to GenBank were KU528848, KU528850, KU528851, KU528852, KU528854, KU528855, KU528857, KU528858, KU528859, KU528860, KU528861, KU528862, and KU528863 (Table 1). Five isolates (21.7%) were identified as *F. oxysporum* and represented by the following accession numbers KU528844, KU528846, KU528847, KU528853, and KU528856. Two isolates (8.7%) were assigned to *F. chlamydosporum* species and were deposited in GenBank with the accession numbers KU528845 and KU528865. Two isolates (8.7%) were affiliated to *F. brachygibbosum* and their representative sequences were deposited in GenBank and assigned KU528849 and KU528864 as accession numbers. Finally, one isolate (4.34%) showed 99% homology with previously published sequences of *F. acuminatum* (GenBank accession No: KU528866). The result of these alignments was in agreement with those of morphological and species-specific PCR studies showed for the 23 sequences 99% homology with previously published sequences of the identified species.

**Fig. 2:**
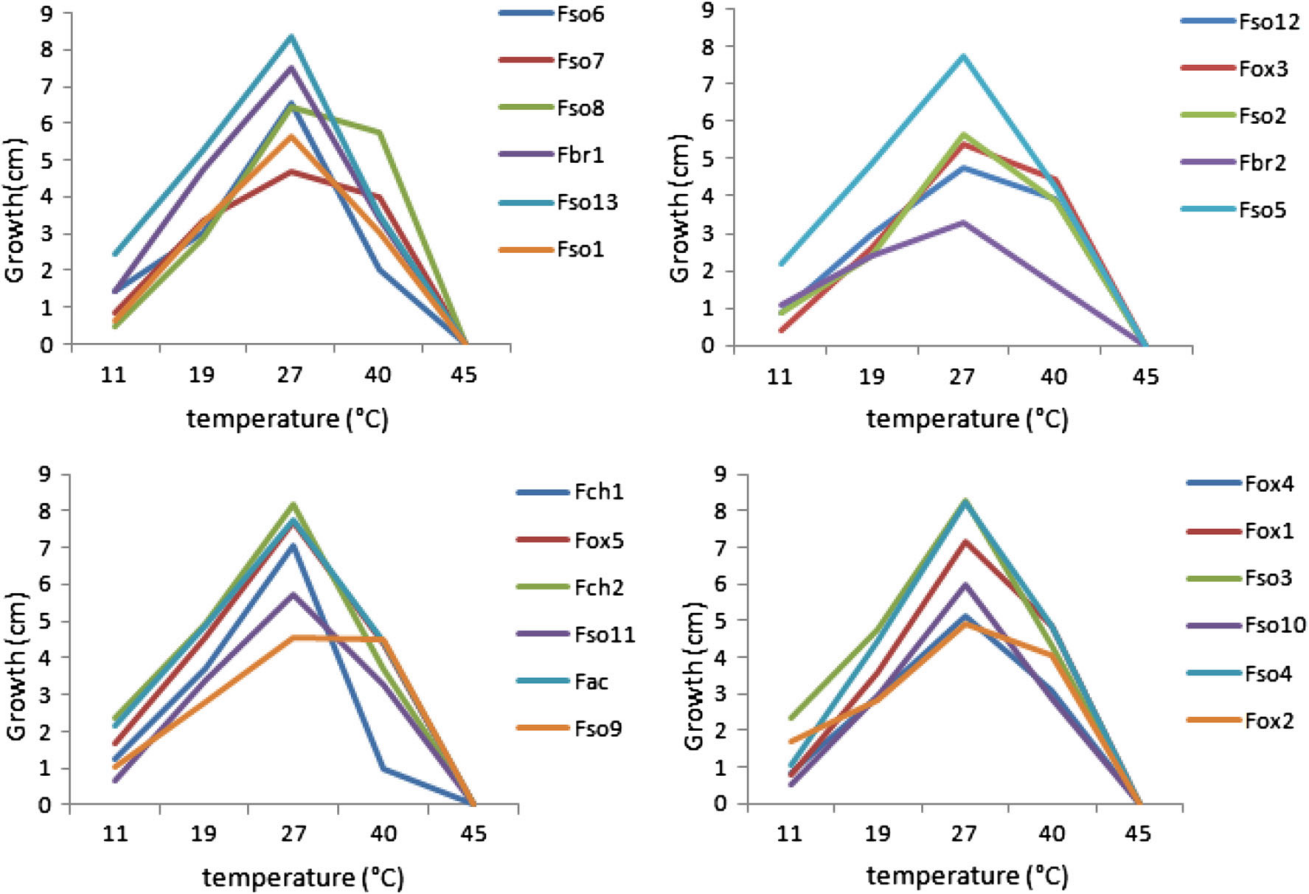
Effect of incubation temperatures on mycelial growth (11, 19, 27, 40 and 45 °C) of the isolates of* Fusarium* spp.;** a** Fso6, Fso8, Fb1, Fso13, Fso1;** b** Fso12, Fox3, Fso2, Fbr2, Fso5;** c** Fch1, Fox5, Fch2, Fso11, Fac, Fso9;** d** Fox4, Fox1, Fso3, Fso10, Fso4, Fox2

The study of the rate of radial growth of the 23 pathogenic *Fusarium* isolates was carried out on two different media SNA and PDA at 27 °C (Table 2). According to data, the radial growth of *Fusarium* spp. was higher on PDA than on SNA medium reaching about 8 mm/day for isolates: Fso3, Fso4, Fso13 and Fch2. Using analysis of variance (ANOVA), no significant difference was found between these four isolates (*P* > 0.05). Data of thermal effect showed a clear variation in the radial growth rate between *Fusarium* species (Fig. 2). For the majority of the isolates tested such as Fso13, Fso4, Fch2, Fac, Fbr1, and Fox1, radial growth reached its maximum at 27 °C. Although, *Fusarium* isolates maintained their level of growth at a temperature of 40 °C. These results confirmed that temperature factor influenced the radial growth of *Fusarium* spp. associated with olive trees. Thus, this parameter depended on the composition of the culture medium and the temperature of incubation.

**Table 2:** Radial growth rate (RGR) on potato dextrose agar (PDA) and Synthetisher Nahrstoff agar (SNA)

Isolates	Means AGR (mm/day) (SNA) ± SD	Means AGR (mm/day) (PDA) ± SD
Fso4	5.507 ± 0.153a	8.290 ± 0.338a
Fac	5.586 ± 0.240a	7.786 ± 0.228bc
Fox2	5.364 ± 0.260a	7.797 ± 0.242bc
Fso3	5.306 ± 0.205ab	8.083 ± 0.370ab
Fso7	5.065 ± 0.585ab	4.550 ± 0.180h
Fso2	4.663 ± 0.197bc	5.878 ± 0.196f
Fso1	4.720 ± 0.251c	5.735 ± 0.236f
Fch1	4.692 ± 0.570c	7.106 ± 0.133d
Fso10	4.258 ± 0.351c	6.006 ± 0.117f
Fox5	4.283 ± 0.123d	5.159 ± 0.213g
Fox4	3.445 ± 0.146e	5.089 ± 0.181g
Fso13	3.607 ± 0.208a	8.107 ± 0.198ab
Fso6	3.484 ± 0.015ef	6.440 ± 0.120e
Fso11	3.310 ± 0.146ef	5.769 ± 0.214f
Fox1	3.327 ± 0.208f	7.309 ± 0.221d
Fbr1	2.631 ± 0.298g	7.481 ± 0.154cd
Fso12	2.366 ± 0.283h	4.926 ± 0.137gh
Fso9	2.244 ± 0.220h	4.856 ± 0.108gh
Fso5	2.012 ± 0.116i	5.152 ± 0.251g
Fbr2	1.652 ± 0.088ij	3.278 ± 0.263i
Fch2	1.386 ± 0.226j	8.191 ± 0.332ab
Fso8	1.174 ± 0.010k	7.797 ± 0.242bc
Fox3	0.670 ± 0.020l	7.468 ± 0.311cd

Data represent mean ± standard deviation (SD) (*n* = 3); (*P *< 0.05)

Fso6, *F. solani* 6; Fso7, *F. solani* 7; Fch1, *F. chlamydosporum* 1; Fox3, *F. oxysporum* 3; Fch2, *F. chlamydosporum* 2; Fso11, *F. solani* 11; Fac, *F. acuminatum*; Fso9, *F. solani* 9; Fso12, *F. solani* 12; Fox5, *F. oxysporum* 5; Fso2, *F. solani* 2; Fbr2, *F. brachygibbosum* 2; Fox2, *F. oxysporum* 2; Fso8, *F. solani* 8; Fbr1, *F. brachygibbosum* 1; Fso13, *F. solani* 13; Fso1, *F. solani* 1; Fox4, *F. oxysporum* 4; Fox1, *F. oxysporum* 1; Fso3, *F. solani* 3; Fso10, *F. solani* 10; Fso4, *F. solani* 4; Fso5, *F. solani* 5

**Fig. 3:**
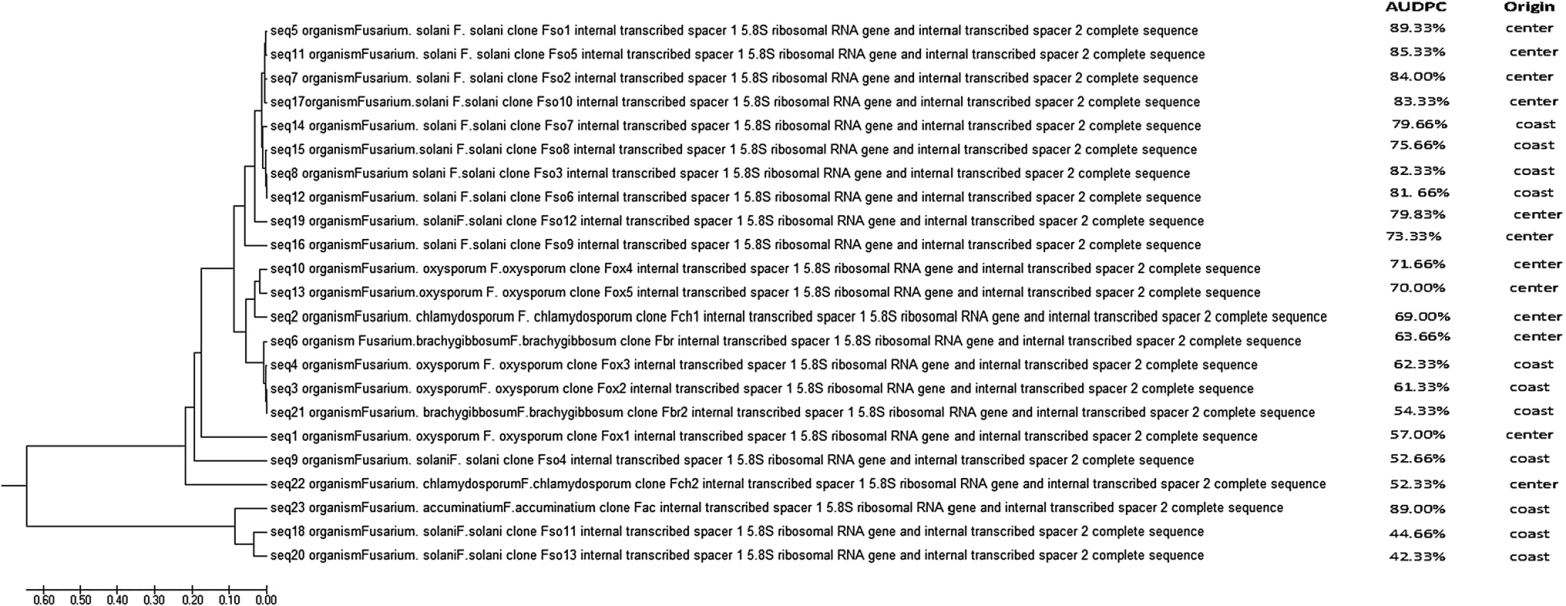
Phylogenetic Tree of pathogenic* Fusarium* isolates

The study of the pathogenicity degree of the 23 *Fusarium* isolates showed significant difference (*P* < 0.05) in their pathogenicity resulting in variable degrees of dieback of the infected plants. 2 months after inoculation, disease incidence reached 42% for the pathogenic isolates causing wilt symptoms compared to control plants. ANOVA results of disease severity were highly variable depending on tested isolates where AUDPC values ranged between 42.72 and 91.84% for pathogenic *Fusarium* isolates. This data indicates an important pathogenicity with varying degrees of aggressiveness (Table 3) . In fact, the isolates Fso1 and Fso2, recovered from Sfax region, and Fso5 from Kasserine site showed the highest virulence level (*P* < 0.05). These isolates were able to kill the plant within 2 months post-infection. Cross sections from plants inoculated by these isolates showed an intense browning of the vascular tissues. However, Fso11 and Fso13 isolates collected from Sousse region were the weakly aggressive isolates causing browning at the basal plant parts only (Fig. 1). The relationship among all isolates of *Fusarium* species, which showed that *F. solani* isolates (Fso1) from central and (Fso13) from coastal regions of Tunisia were placed in separated lineage with varied pathogenicity (Fig. 3).

**Table 3:** Results of pathogenicity test of *Fusarium* spp. isolates, data represent mean ± standard deviation (SD) (*n* = 3); (*P* < 0.05)

Isolates	Means AUDPC ± SD	LSD^a^
Fso1	89.33 ± 2.082	a
Fac	89 ± 1	a
Fso5	85.33 ± 2.082	b
Fso2	84 ± 2	bc
Fso3	82.33 ± 2.517	cd
Fso6	81.667 ± 1.528	cd
Fso7	79.667 ± 0.577	d
Fso8	75.667 ± 1.528	e
Fso9	73.333 ± 0.577	ef
Fox4	71.667 ± 1.528	fg
Fox5	70 ± 1	gh
Fch1	69 ± 1	h
Fbr1	63.66 ± 1.528	i
Fox3	62.667 ± 1.528	i
Fox2	61.333 ± 1.155	ij
Fso12	60 ± 1	j
Fox1	57 ± 1.732	k
Fbr2	54.333 ± 1.528	kl
Fso4	52.667 ± 3.055	l
Fch2	52.33 ± 2.082	l
Fso10	47 ± 2.646	m
Fso11	44.667 ± 2.309	m
Fso13	42.333 ± 0.5777	n

Fac, *F. acuminatum*; Fbr 1, *F. brachygibbosum* 1; Fch1, *F. chlamydosporum* 1; Fso1, *F. solani* 1; Fso2, *F. solani* 2; Fso3, *F. solani* 3; Fso4, *F. solani* 4; Fso5, *F. solani* 5; Fox1, *F. oxysporum* 1; Fox2, *F. oxysporum* 2; Fox3, *F. oxysporum* 3; Fso6, *F. solani* 6; Fso7, *F. solani* 7; Fox4, *F. oxysporum* 4; Fox5, *F. oxysporum* 5; Fso8, *F. solani* 8; Fso9, *F. solani* 9; Fso10, *F. solani* 10; Fso11, *F. solani* 11; Fso12, *F. solani* 12; Fso13, *F. solani* 13; Fbr2, *F.* *brachygibbosum* 2; Fch2, *F. chlamydosporum* 2

^a^ *P* < 0.05

It should be indicated that 10–20% of sampling fields showing wilting disease were previously cropped with Solanaceous species. About 84.61% of *F. solani* isolates were recovered from samples collected from orchards already cropped with pepper, potato and tomato (Table 1).

## Discussion

*Fusarium* is one of the important phytopathogenic genera associated with dieback symptoms of olive trees. Our survey clearly demonstrated that 104 of isolates recovered from 150 samples collected from olive trees showing dieback and wilting symptoms in various olive-growing regions in Tunisia were identified as *Fusarium* spp. Khosrow et al. (2015) have also reported similar results in Malaysia.

Few studies have investigated the pathogenicity of *Fusarium* spp. of the olive. Many *Fusarium* isolates were considered as weak or opportunistic as they are able to attack only the plants already weakened by other abiotic stress such as drought, wind, and insect pests (Palmer and Kommedahl 1960). In the present study, the pathogenicity test performed on young olive trees (cv. Chemlali), demonstrate that only 23 out of 104 *Fusarium* spp. isolates collected were pathogenic. The species of *F. brachygibbosum* and *F. chlamydosporum* have not yet been published in Tunisia as pathogens of olive trees. Cross sections from plants inoculated by these isolates showed an intense browning of the vascular tissues. The other isolates were weakly or not pathogenic as they caused browning at the basal plant parts only. According to Van Alfen (1989), browning of the vascular tissues is a result of physical blockage due to the rotted roots and root rotting which is also a disease symptom.

Our observations in different Tunisian olive groves have shown that disease severity can be increased under certain environmental conditions especially in spring with average temperatures varying between 25 and 30 °C, in flooding and in irregular irrigation furrow. The majority of *Fusarium* isolates grown on PDA medium reached their maximum radial growth rates at 27 °C. The activity of soilborne fungi is generally controlled by temperature among other biotic and abiotic factors (Theron and Holz 1990; Brock et al. 1994; Dix and Webster 1995).

Our data clearly demonstrated that the radial growth of *Fusarium* spp. was higher on PDA than on SNA medium. Under natural conditions, fungus growth is very slow. This is due to the low intake of substrates and to the heterogeneity of nutrient distribution in microbial habitats. Growth can also be slowed by other factors such disturbances as antagonistic interactions with other species which compete for the same substrate, animal invasions, the stress caused by nutrient depletion and changes in physical conditions (temperature, pH, etc.) (Brock et al. 1994).

Severity and incidence of diseases caused by each *Fusarium* species vary according to the geographical location, climatic factors and cultural practices (Daami-Remadi 2006). Different fungal pathogens were isolated with varying frequencies, but the most frequently isolated fungi from rotten roots and crowns were *Fusarium* spp. These fungi were consistently isolated from contaminated cuttings of olive trees removed from different areas, suggesting their potential involvement in the spread and incidence of root rot disease. In addition, in Tunisia, many farmers produce the olive through vegetative cuttings suggesting that these pathogens have been broadcasted from a producing area to another as reported from other countries (Rodriguez et al. 2008). Our results confirm this observation. In fact, 84.61% of *F. solani* isolates were recovered from samples collected from orchards already precultivated by pepper, potato and tomato crops. Intercropping of Solanaceous crops within olive groves may be a source of transmission of fungal diseases as these crops are very susceptible to soilborne pathogens including *Fusarium* spp. and *V. dahliae* (Serrhini and Zeroual 1995; Gharbi et al. 2015). This is because many of these crops (cotton, potato, tomato, alfalfa, or even olive itself) increase the pathogen population in soil in a very efficient way (Tjamos and Tsougriani 1990; Bejarano-Alcázar et al. 1996). The severity of *Fusarium* diseases is conditioned by various factors, including the climate conditions and the physiology of the host plant (Tivoli et al. 1986).

In conclusion, the results of present study stated that *Fusarium* spp. might be a major agent causing wilt and dieback of olive trees in Tunisia. Although further studies on cross pathogenicity by predicting the virulence of isolates collected from other crops towards olive will provide crucial information to be used for rotations of the annual crops that are grown within or adjacent to olive orchards. This information will help the farmers to decide which crops may not be suitable for their fields.
